# Implant Placement in the Palatal Root Socket of Maxillary Molars to Avoid Posterior Cantilevers in All-on-X Treatments

**DOI:** 10.7759/cureus.96476

**Published:** 2025-11-10

**Authors:** Pedram Yaghmai, Gregori M Kurtzman

**Affiliations:** 1 Oral Surgery, Private Practice, Burke, USA; 2 Dentistry, Private Practice, Silver Spring, USA

**Keywords:** all-on-x, cantilever, maxillary arch rehabilitation, palatal root extraction socket, pterygoid implants, sinus augmentation alternative

## Abstract

The treatment of the maxillary arch for an All-on-X approach can be challenging due to sinus enlargement, necessitating the use of pterygoid implants to avoid the creation of a cantilever on the prosthesis. Posterior prosthesis cantilevers may lead to problems such as screw loosening or fracture, as well as bone loss on the anterior implants due to the posterior loads on the cantilever. When maxillary molars are present and would be extracted as part of the All-on-X approach, utilizing the palatal extraction socket for immediate implant placement allows for the avoidance of sinus augmentation and potential complications associated with the surgical placement and restoration of pterygoid implants, while also eliminating the prosthetic cantilever. This report of two cases examines the issues related to prosthetic cantilevers and describes the surgical process of immediate implant placement using maxillary molar palatal root extraction sockets.

## Introduction

Implant treatment of the edentulous maxilla can present clinical challenges related to the available bone following crestal bone resorption and sinus enlargement that occur after edentulation of the arch [1]. Patients may present with full maxillary dentures seeking an implant solution to provide a more stable dentition with an All-on-X approach. Treatment to facilitate implant placement in these clinical situations involves sinus augmentation via either a crestal or lateral approach, depending on the available crestal height. Zygomatic implants and pterygoid implants have been employed to avoid sinus augmentation and the delays in treatment completion that often accompany extensive grafting to enable implant placement in these sites [2].

Treatment of a maxilla with failing dentition also poses similar challenges. Crestal bone loss is common in these patients, leading to periodontal issues that necessitate the replacement of those teeth with implants for an All-on-X treatment approach. Maxillary sinus enlargement is often noted, and cone-beam computed tomography (CBCT) cross-sectional views of the molars frequently demonstrate the buccal roots in minimal bone, while the palatal root is found in the denser bone of the palate. Employing the palatal root socket at extraction for implant placement in an All-on-X approach can allow for the avoidance of sinus augmentation, thereby shortening treatment time [3]. This can enable immediate provisional hybrid prosthetic restoration placement at the time of surgery, provided adequate implant insertion torque is achieved.

In traditional All-on-X treatment, a posterior cantilever is commonly present, as implant placement does not occur in the molar area due to insufficiently available crestal height related to sinus enlargement. In such cases, implants are confined to the premolar area and anterior to that. Posterior cantilevers can face issues over time due to increased load on the unsupported molar area of the prosthesis. As occlusal load increases toward the tuberosity area of the ridge, a posterior cantilever will undergo higher stress and load than areas anterior to it. It has been reported that the implant survival rate tends to be lower in cases with a posterior cantilever, and marginal bone loss around anterior implants tends to be higher [4]. Additionally, the incidence of mechanical complications significantly increases. Cantilevers increase the probability of failure of the prosthesis-abutment complex, as they increase stresses on the structures compared to studies conducted without cantilevers [5,6]. Common complications reported include screw loosening and/or porcelain fracture. Increased off-axis loading on the prosthetic screw in these cantilever cases may lead to screw loosening or screw fracture [7].

Therefore, reducing the cantilever is essential for the long-term success of both the prosthesis and the implants. Pterygoid implants have been employed to eliminate this cantilever. If available, utilizing the palatal root socket can also serve as a site for implant placement, which can reduce or eliminate the cantilever. However, pterygoid implants can present complications such as bleeding, edema, infection, trismus, pain, and neurosensory disturbances. There is also the risk of implant displacement into the pterygoid fossa or sinus membrane perforation in atrophic maxillary cases [8]. Furthermore, the surgical placement of pterygoid implants, as well as the restoration of those implants due to access, poses challenges for both the surgeon and the restoring dentist.

A study reported that maxillary molars, 7% of the first and 4% of the second molars, had alveolar anatomy adequate for immediate implant placement when the implant is centered in the extraction socket [9]. Another study indicated that 70% of maxillary first molars were in contact with the maxillary sinus. The palatal root demonstrated an average of one-fifth of its root surface in contact with the sinus, whereas the mesio-buccal was one-sixth and the distal-buccal was somewhat less in contact with the sinus. This resulted in the palatal root having a contact area with the maxillary sinus of 27.8 ± 21.4 mm² (20% of the root area), followed by the mesio-buccal at 20.5 ± 17.9 mm² (17% of the root area), and the distal-buccal root at 13.7 ± 12 mm² (14% of the root area) [10]. This may limit immediate implant placement into molar extraction sockets when parallel implants are placed anterior to them. Immediate implant placement into the palatal root socket of maxillary molars simplifies treatment, resulting in shorter treatment times compared to sinus augmentation for implant placement. Additionally, surgical trauma can be minimized, and complications associated with sinus augmentation surgery or pterygoid implant placement can be avoided. A recently reported case involving palatal root socket placement and follow-up over two years indicated clinical success with no complications [3].

Osseodensification aids in improving bone density in the maxillary arch with any planned implant placement across the arch. This is especially beneficial with immediate implant placement in the palatal extraction socket, as it decreases bone removal that occurs with standard osteotomy drills. The improvement in bone density results in increased implant stability at placement (higher insertion torque) when utilizing osseodensification burs [11]. Following the extraction of the tooth and curettage of the palatal root socket, after confirming that no sinus perforation is present, a pilot drill may be utilized if additional length is required for the implant. The osseodensification burs are used to avoid standard osteotomy drills, helping to preserve the existing bone and prevent sinus perforation. Short implants (6-8 mm) may be utilized; however, it is recommended that wider diameter implants (5 mm or greater) be selected in these clinical situations to provide sufficient bone-to-implant (BOI) contact to manage the loading of posterior implants under function.

As with all techniques, patient selection is key to clinical success. CBCT analysis, specifically cross-sectional views of the maxillary molars, aids in determining the bone present around the palatal root and whether sufficient osseous thickness exists between the buccal aspect of the root and the sinus. Osseodensification helps maintain the bone and improves its density to better stabilize the implant at placement and manage loading when prosthetically loaded. The technique carries similar risks to crestal sinus lift procedures and requires surgical experience with implant placement in close proximity to the maxillary sinus.

Two clinical cases are presented in this report illustrating the use of the palatal root socket at the time of edentulation of the arch to avoid sinus augmentation or the use of zygomatic or pterygoid implants.

## Case presentation

Case 1

**Figure 1:**
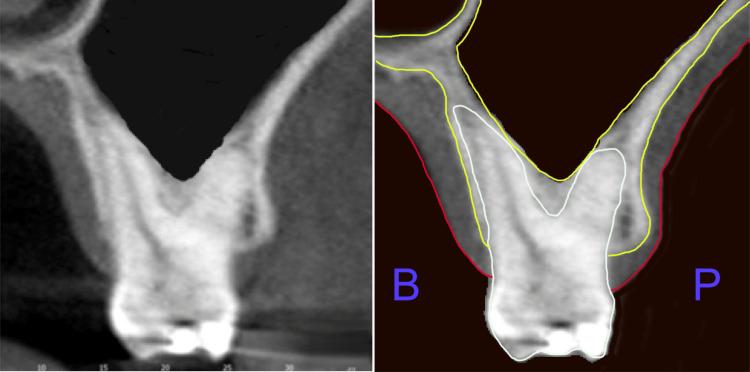
CBCT cross-section of the maxillary molar demonstrating minimal bone on the buccal roots related to sinus enlargement, with the palatal root remaining within bone. The left panel shows the unmarked CBCT slice, while the right panel includes markings to highlight key features. CBCT: Cone-beam computed tomography

**Figure 2:**
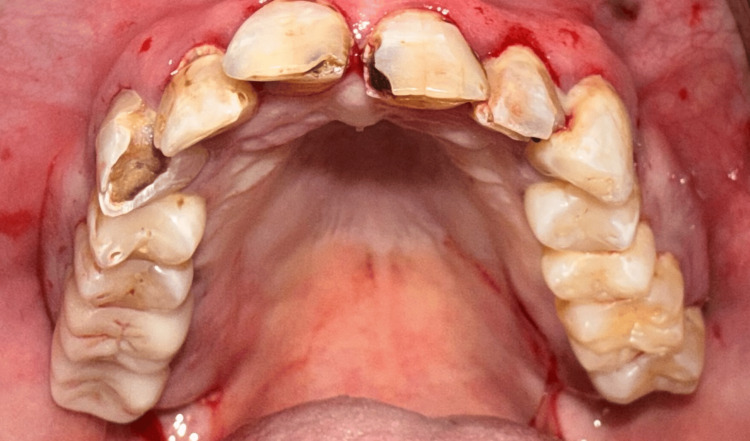
Maxillary dentition failing due to extensive decay and periodontal issues.

A 43-year-old female patient presented with failing dentition in the maxillary and mandibular arches. The patient expressed a desire for an implant treatment approach, not wishing to wear full dentures as had been recommended as a treatment option. The examination noted generalized caries and periodontal bone loss in the dentition (Figures 1-2). Full dentition was present in both arches.

**Figure 3:**
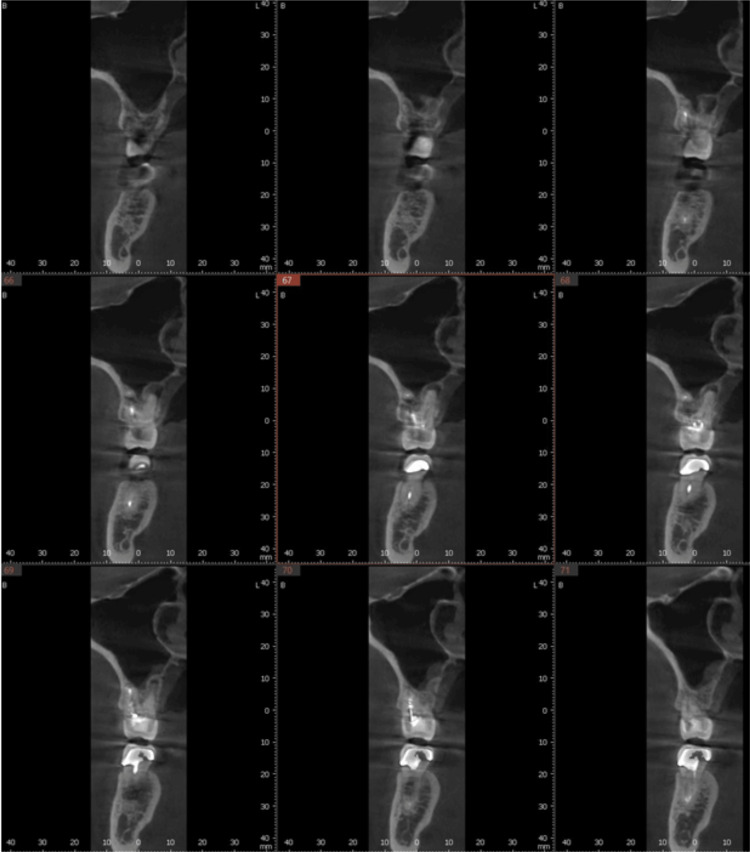
Preoperative CBCT scan of the maxillary right first molar evaluating available bone for planned implant placement at the failing tooth site. CBCT: Cone-beam computed tomography

**Figure 4:**
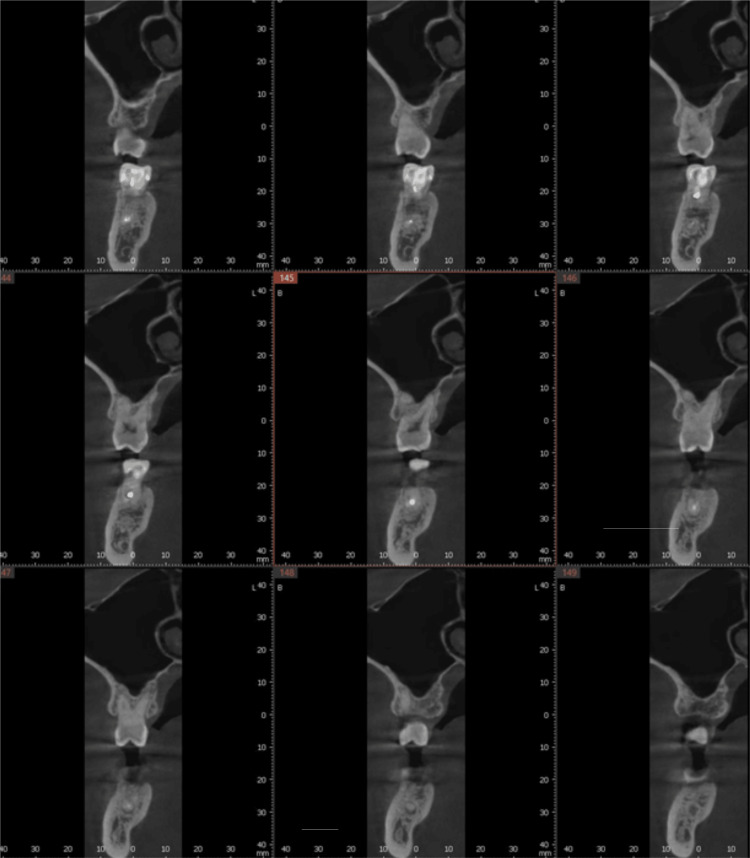
Preoperative CBCT scan of the maxillary left first molar evaluating available bone for planned implant placement at the failing tooth site. CBCT: Cone-beam computed tomography

A CBCT was taken to evaluate anatomical structures and potential implant placement. The preoperative CBCT noted bilateral enlargement of the maxillary sinuses, with the buccal roots of the molars in minimal bone due to that enlargement (Figures 3-4). The palatal roots of the molars were found to be situated in bone. A treatment plan was formulated to extract the failing dentition and place implants in both arches for an All-on-X approach. In the maxillary arch, following edentulation, the bone crest would be reduced and implants would be placed bilaterally at the lateral incisors, first premolars, and palatal root of the first molars. The mandibular arch would also receive six implants, so both arches would be treated with an All-on-6 approach. Restoration would be done with a fixed hybrid prosthesis in both arches. This treatment plan was discussed with the patient, and any questions she had were answered. She accepted the presented treatment plan and was scheduled for the surgical appointment.

**Figure 5:**
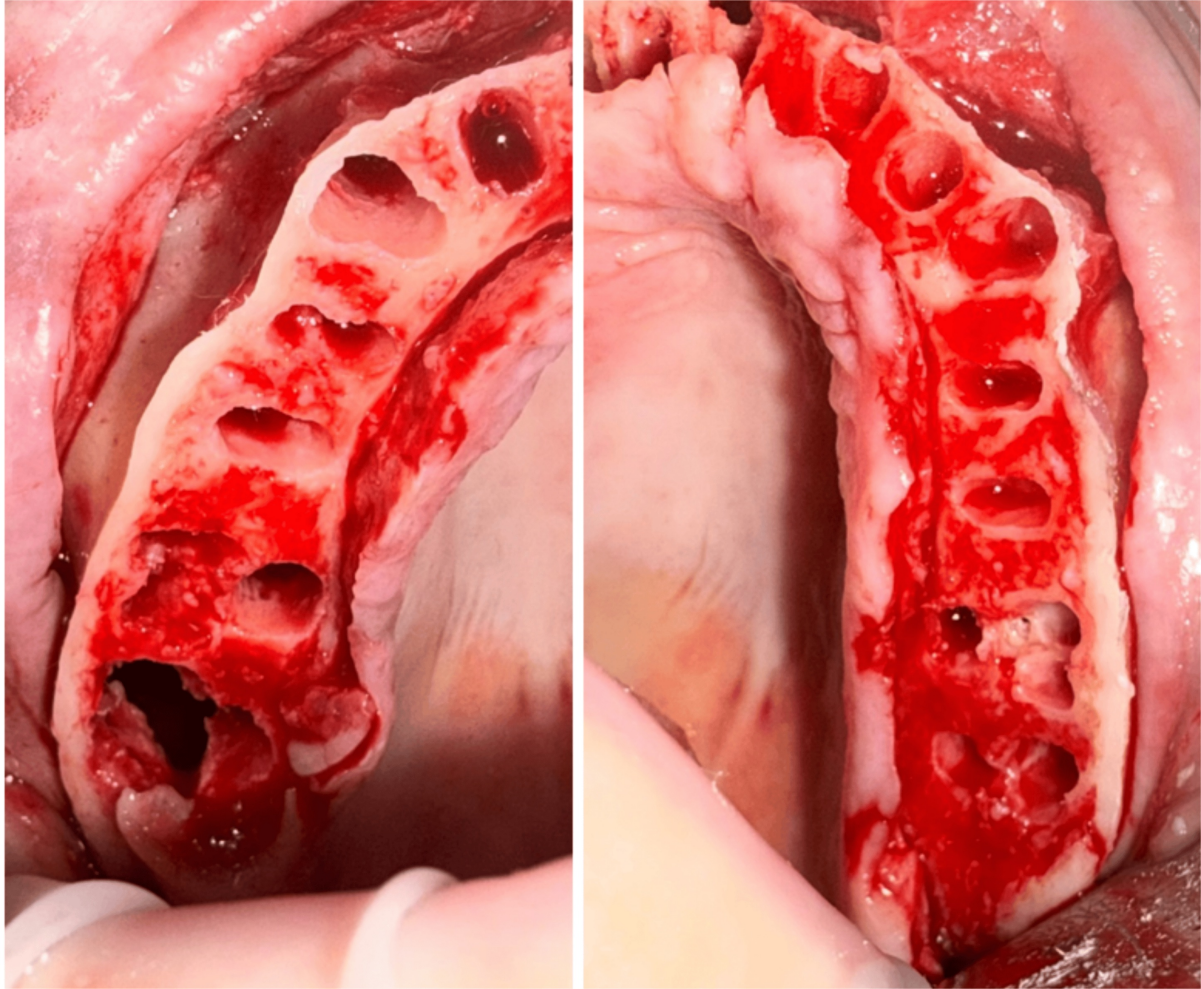
Maxillary arch following edentulation and crestal reduction in preparation for implant placement. Note the sinus communication at the right second molar extraction socket. The left panel corresponds to the maxillary right quadrant, and the right panel corresponds to the maxillary left quadrant.

**Figure 6:**
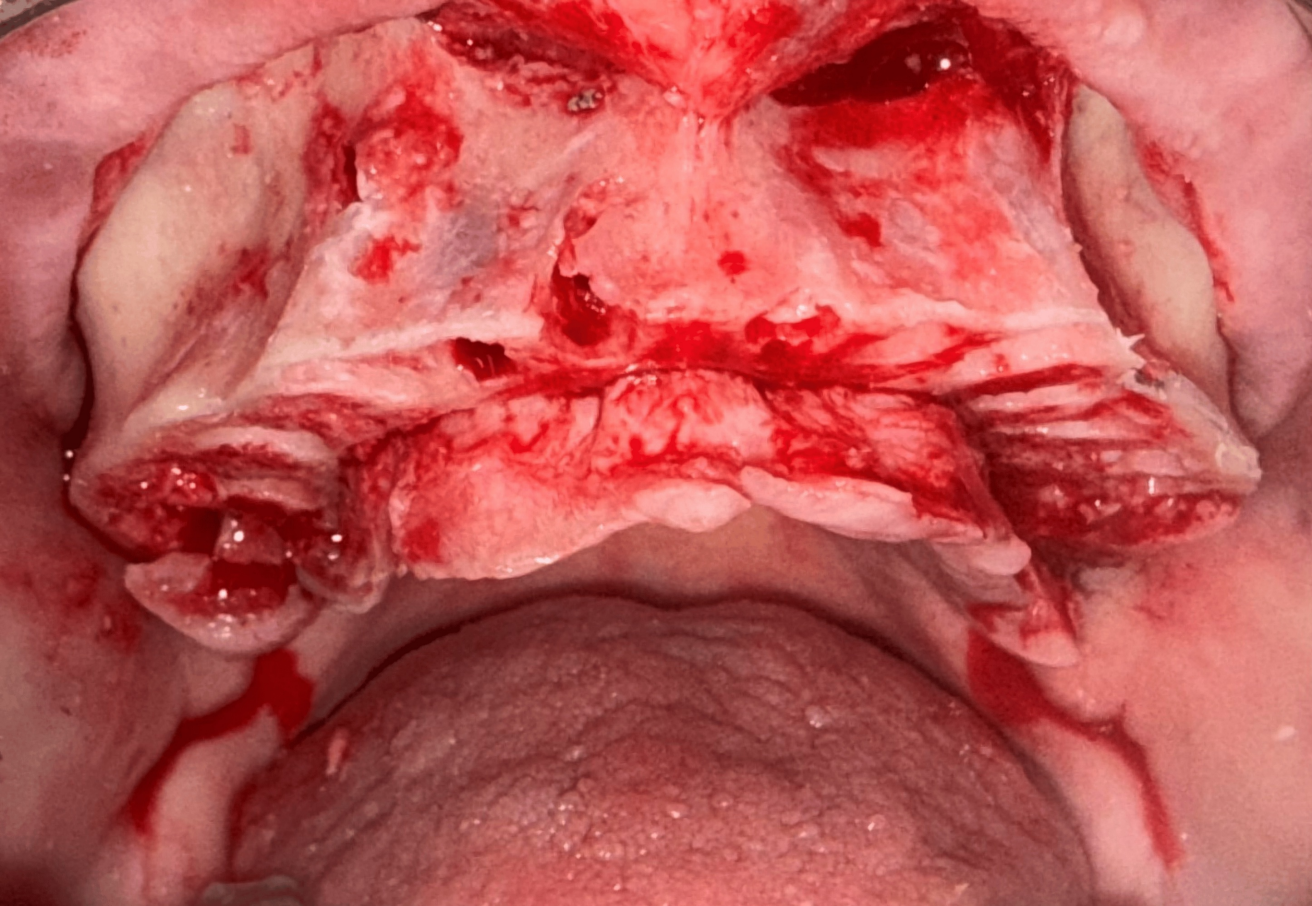
Maxillary arch following edentulation and crestal reduction in preparation for implant placement.

The patient presented for the surgical appointment, and the consent form was reviewed and signed by her. The patient was sedated with IV anesthesia, and a local anesthetic was administered in both arches. The dentition in both arches was extracted utilizing elevators and forceps. Following edentulation, a crestal incision was made from tuberosity to tuberosity, with no vertical releasing incisions, and the maxillary arch was flapped to expose the entire arch. Crestal bone reduction was made, reducing the ridge by 5 mm to provide a flat, wide ridge in preparation for implant placement (Figures 5-6). A sinus exposure was noted at the extraction socket for tooth #2, the right second molar.

**Figure 7:**
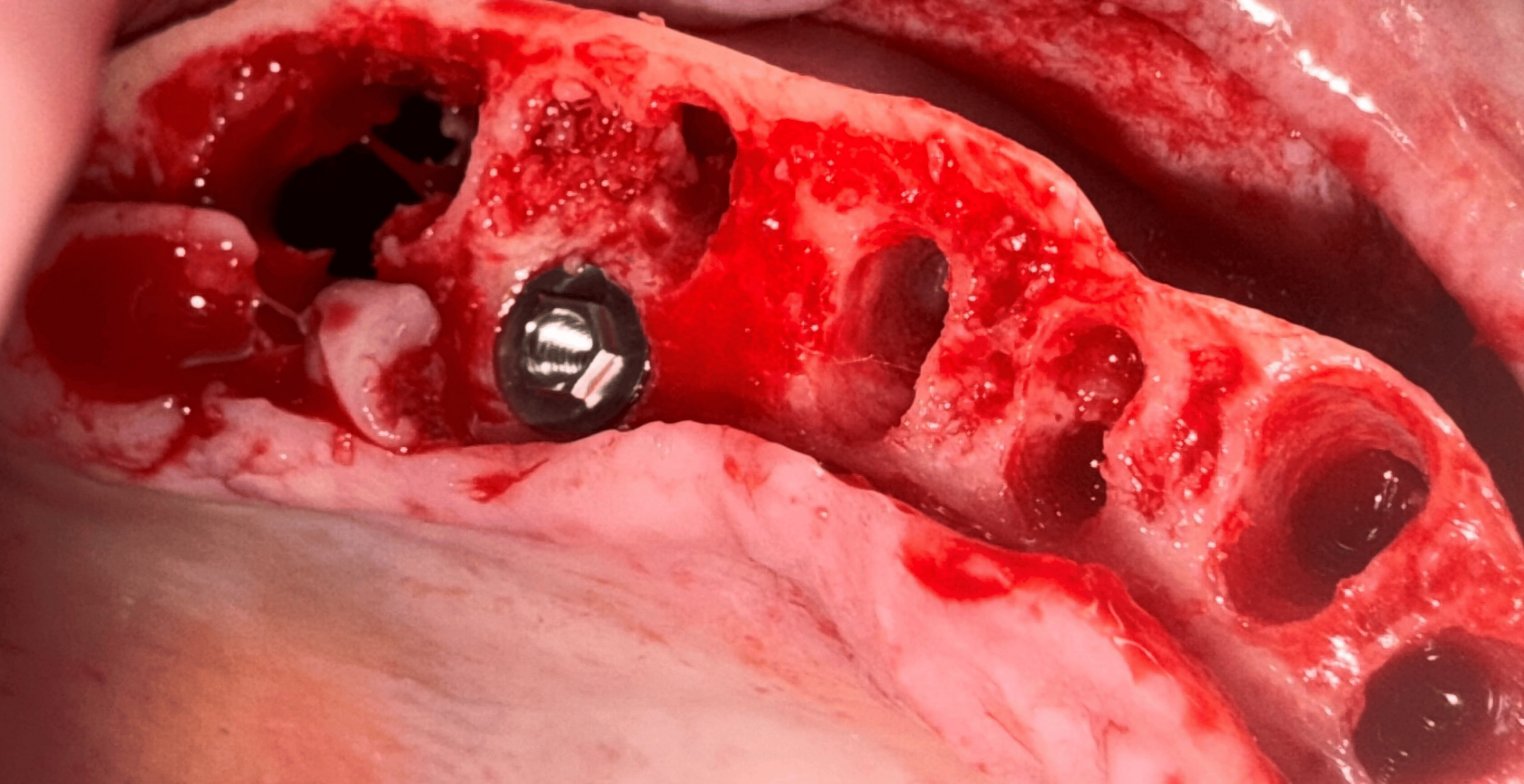
Following edentulation of the maxillary arch and crestal bone reduction, an implant was placed into the palatal root socket of the right first molar.

**Figure 8:**
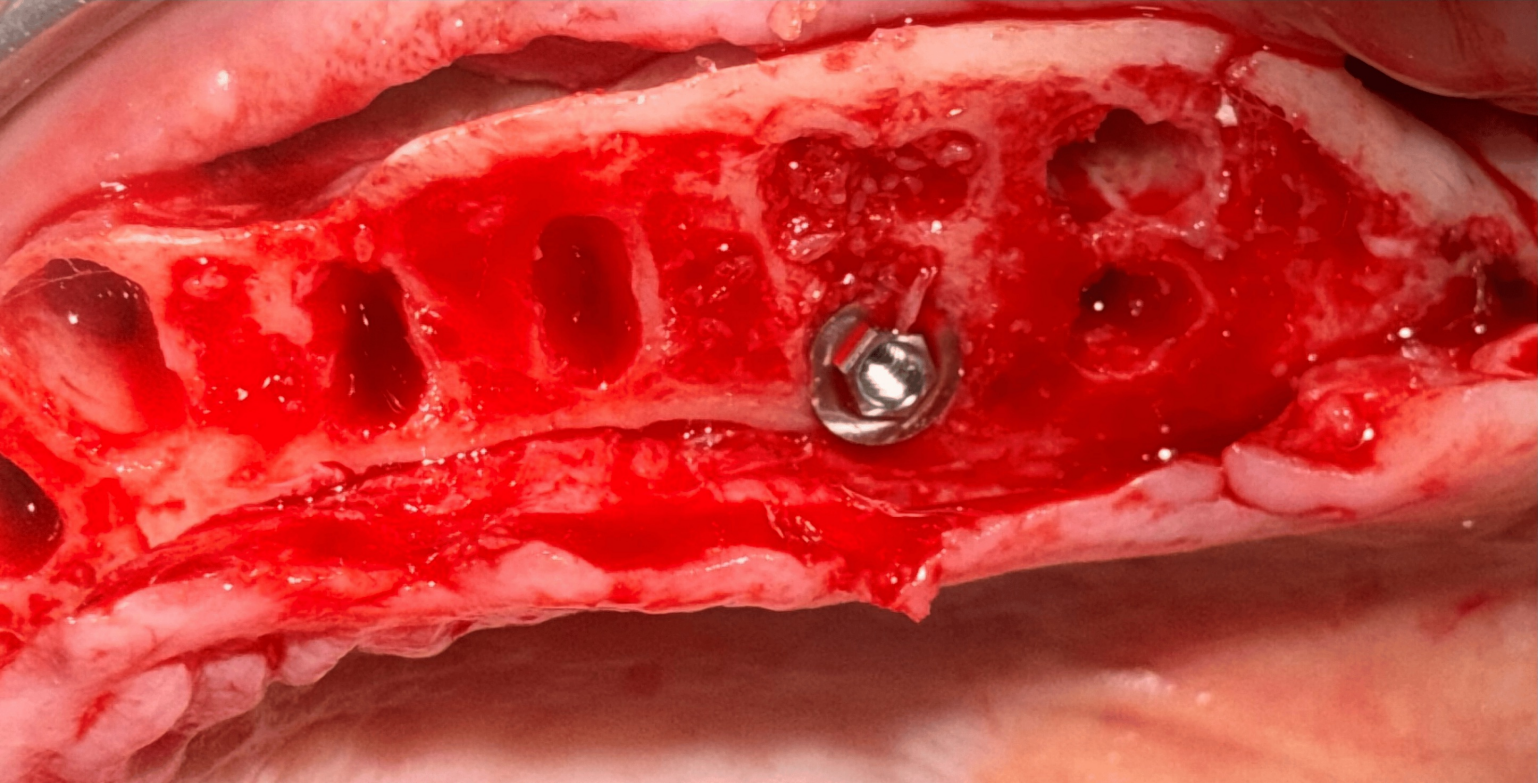
Following edentulation of the maxillary arch and crestal bone reduction, an implant was placed into the palatal root socket of the left first molar.

**Figure 9:**
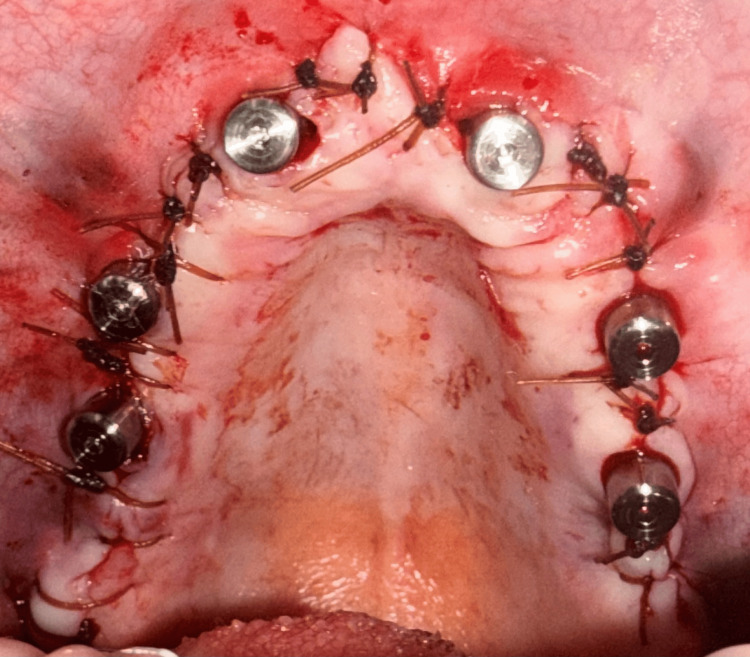
Maxillary arch following implant placement and soft tissue closure around the healing abutments.

Osteotomies were made at the palatal socket of the first molars bilaterally. To avoid potential sinus perforation during site preparation, traditional implant drills were not utilized. The palatal root socket was treated with Densah® osteodensification drills (Versah, Jackson, MS, USA) to lift the sinus from a crestal approach and laterally expand the socket while improving bone density. Implant width in these palatal root sites is usually 4 or 5 mm in diameter, though in some cases, a 6 or 7 mm diameter implant may be necessary to obtain initial stability. The goal is to achieve an insertion torque of 30 Ncm or greater at placement to allow immediate placement of a provisional prosthesis. A 5 mm x 8.5 mm implant (External Tapered, Southern Implants, Jupiter, FL, USA) was placed into the osteotomy at the #3 palatal root socket (Figure 7). This was followed by the placement of a 5 mm x 8.5 mm implant (External Tapered) into the osteotomy at the #14 palatal root socket (Figure 8). Additionally, implants were placed at sites #5 (5 mm x 10 mm) (External 12° Co-Axis® Implant, Southern Implants, Jupiter, FL, USA), #7 (5 mm x 11.5 mm) (External 12° Co-Axis® Implant), #10 (5 mm x 11.5 mm) (External 12° Co-Axis® Implant), and #12 (5 mm x 10 mm) (External 12° Co-Axis® Implant) following site preparation. The insertion torque for all implants was 50 Ncm. Straight mult-unit abutments (MUAs) (Southern Implants, Jupiter, FL, USA) were placed on the implants, including the Co-Axis® implants. Healing abutments (Southern Implants, Jupiter, FL, USA) were placed on the implants in the maxillary arch, and the flap was re-approximated to achieve primary closure. Sutures were placed to secure the flap margins (Figure 9).

**Figure 10:**
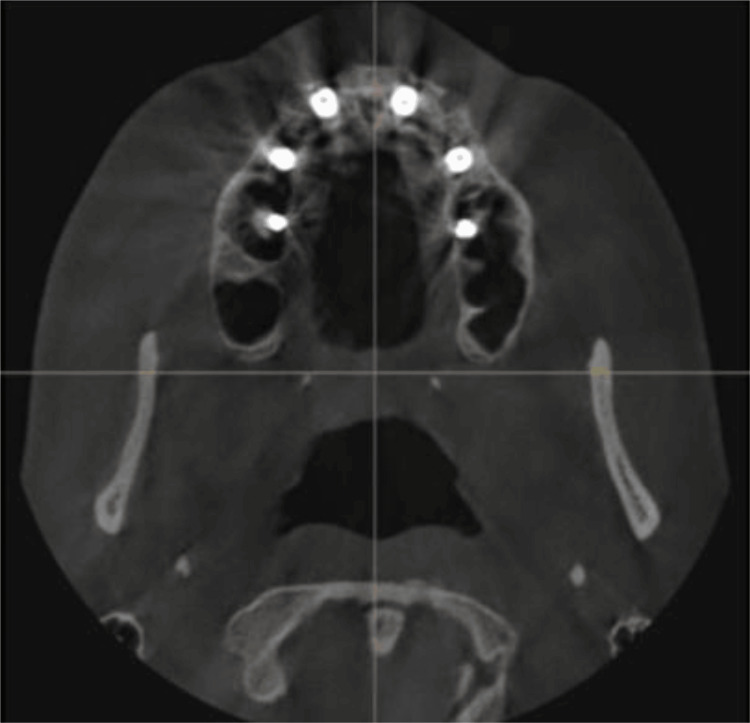
Post-implant CBCT view of the maxillary implants demonstrating implant positioning in relation to the buccal and palatal aspects of the ridge. CBCT: Cone-beam computed tomography

**Figure 11:**
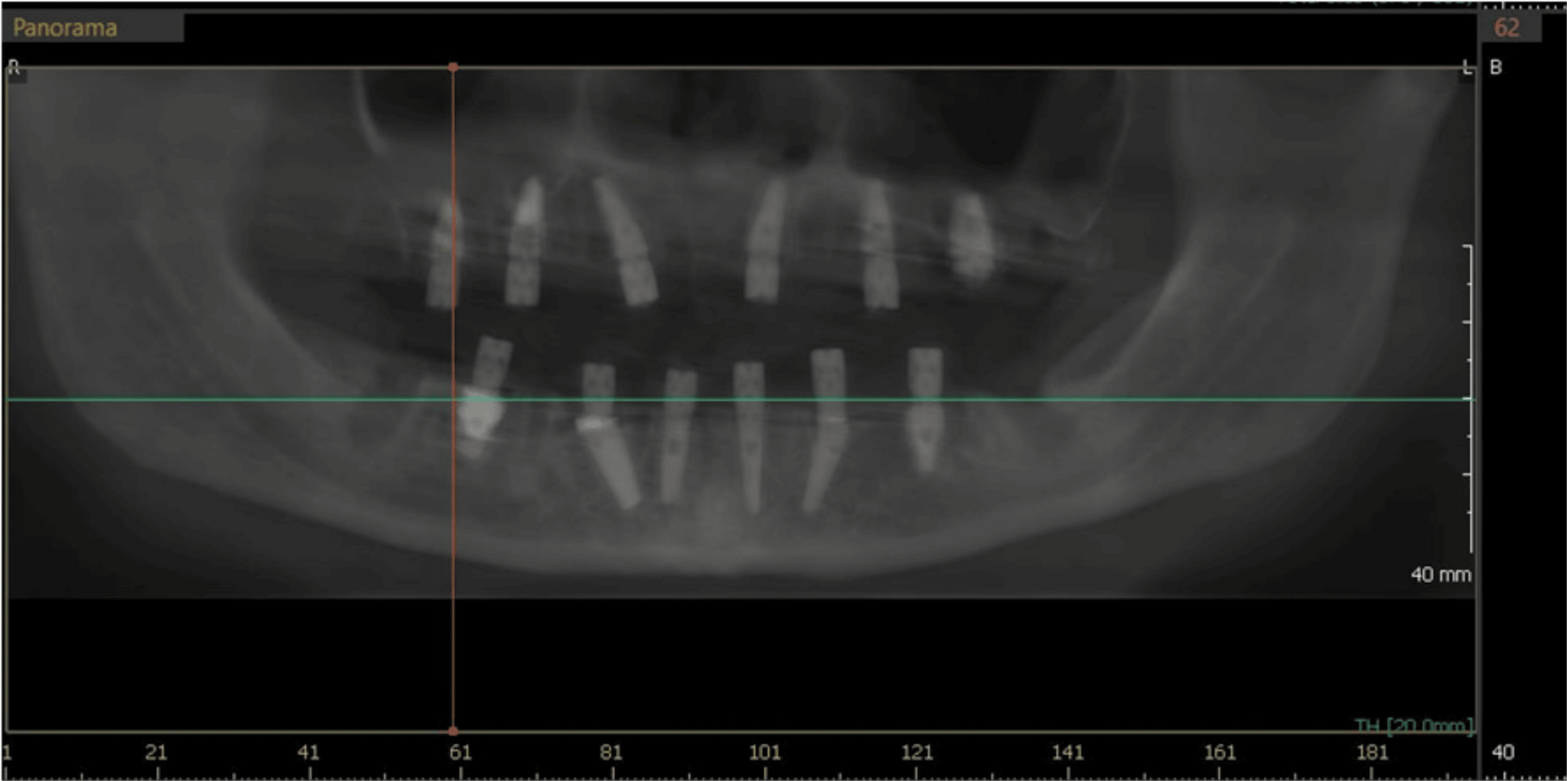
CBCT panoramic view showing implants placed in both arches and their relation in the maxillary arch to the sinus and nasal fossae. CBCT: Cone-beam computed tomography

**Figure 12:**
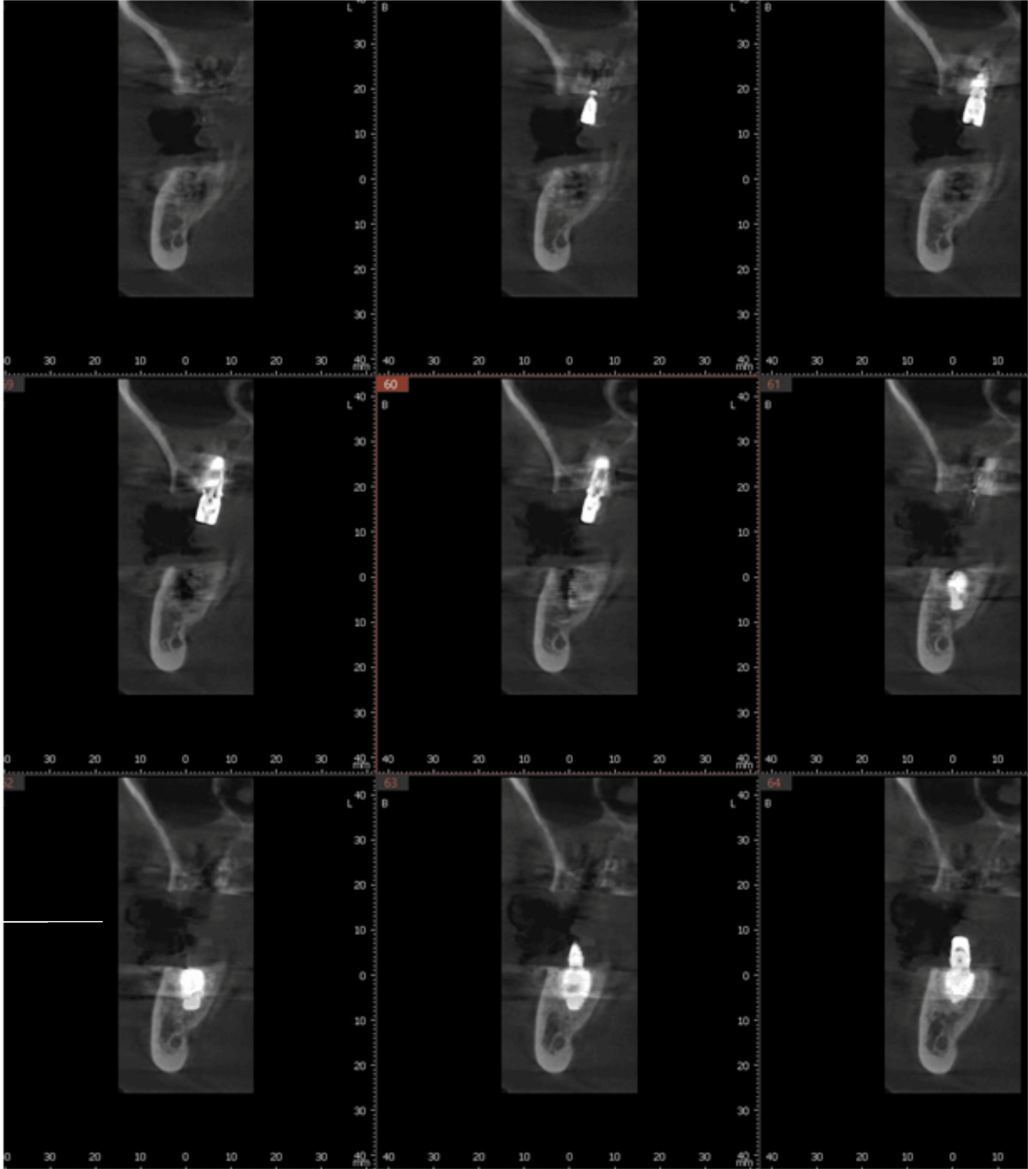
CBCT scan of the implant placed into the palatal root socket of the right first molar in relation to the maxillary sinus. CBCT: Cone-beam computed tomography

**Figure 13:**
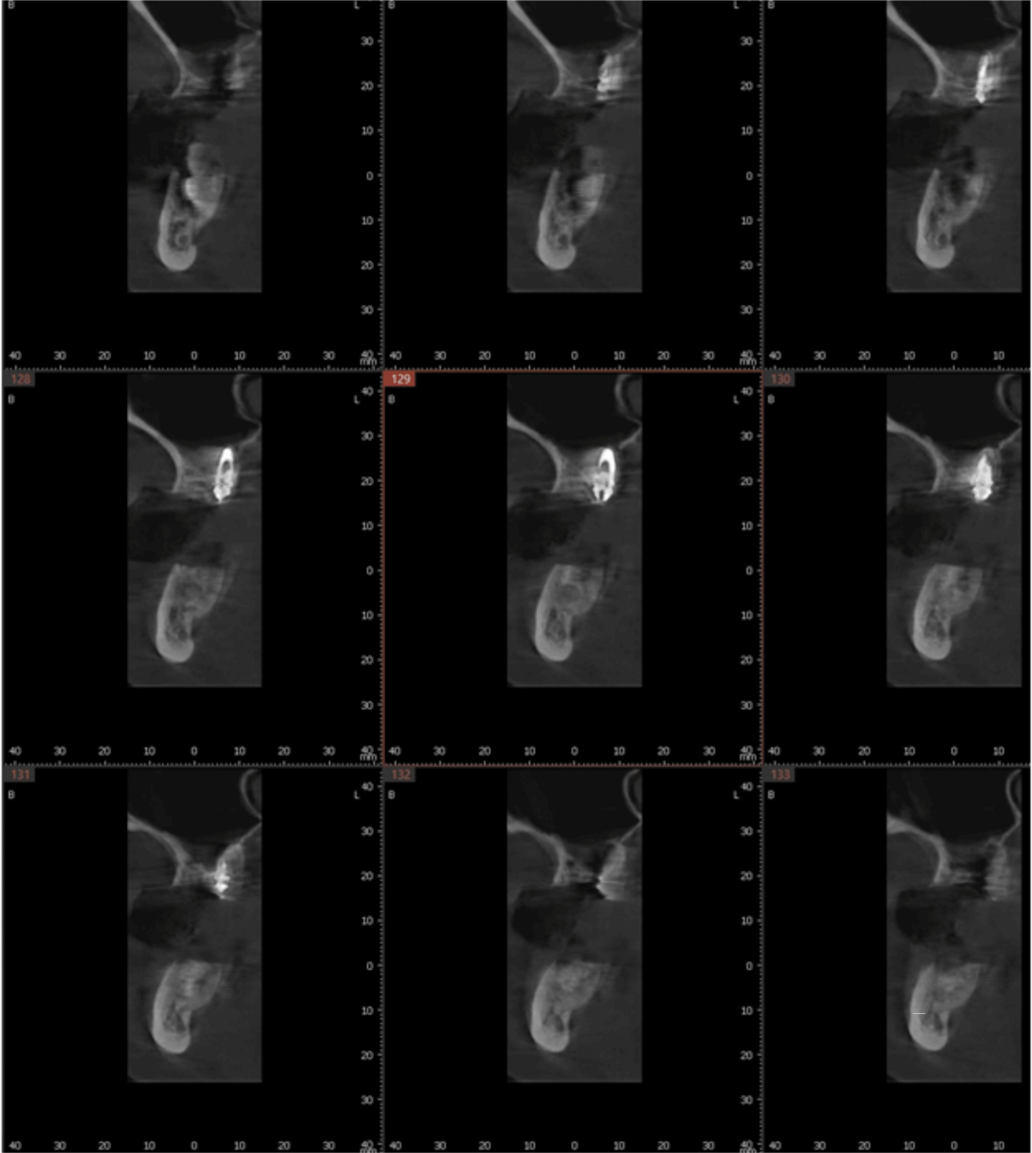
CBCT scan of the implant placed into the palatal root socket of the left first molar in relation to the maxillary sinus. CBCT: Cone-beam computed tomography

A post-surgical CBCT was taken. The occlusal view demonstrated that the implants were within the buccal and palatal ridge plates (Figure 10). The panoramic view noted that the posterior implants were not in the maxillary sinus, and the anterior implants avoided the nasal fossae at placement (Figure 11). The cross-sectional views of the implants placed at the first molars bilaterally into the palatal root sockets demonstrated the implants within bone, with no sinus perforation, avoiding the sinus buccal to the implant position (Figures 12-13).

**Figure 14:**
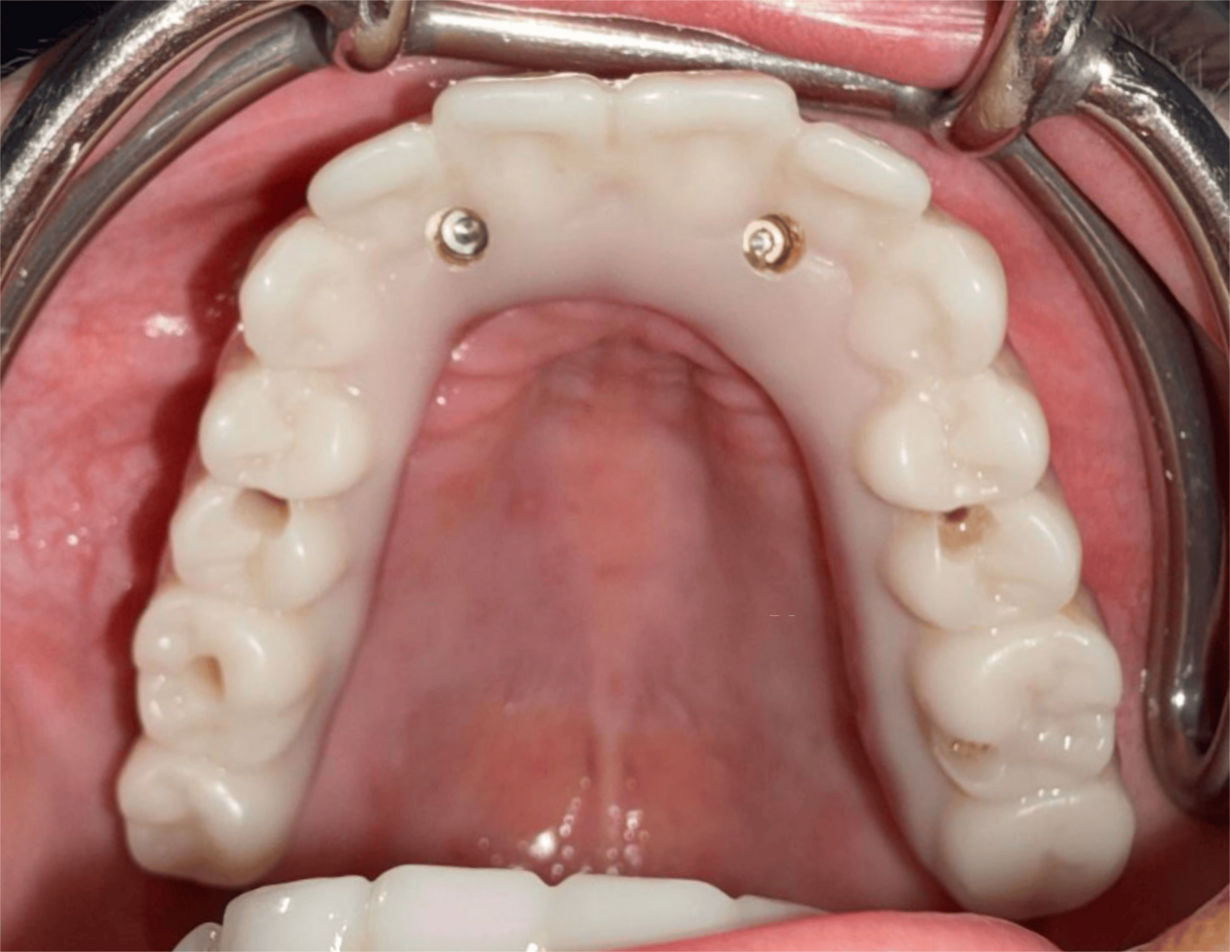
Occlusal view of the maxillary provisional hybrid prosthesis demonstrating the achieved AP spread. AP: Anteroposterior

An immediate provisional hybrid prosthesis was delivered on the day of surgery (Figure 14). Polymethyl methacrylate (PMMA) provisionals were made ahead of time, and EasyPro packs (Keystone Dental, Burlington, MA, USA) were used for intraoral pickup for immediate conversion by the prosthodontist.

Case 2

A 58-year-old male patient presented with failing dentition in the maxillary arch. Clinical examination revealed that failing restorations on teeth #14, #15, and #13 were missing. Based on the condition of the maxillary dentition, it was recommended that the patient undergo edentulation of the arch, placement of implants, and a full arch restoration utilizing an All-on-X approach, as this was deemed the most affordable option due to the presence of many missing/non-restorable teeth in the maxilla.

**Figure 15:**
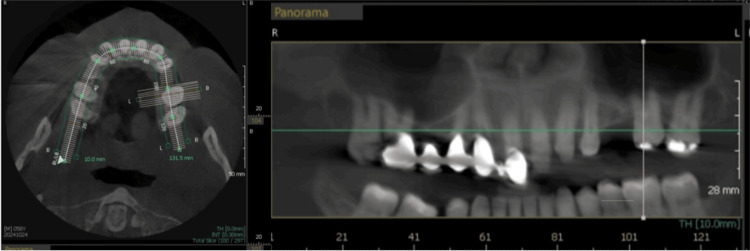
CBCT scan evaluating the failing maxillary dentition in relation to the sinus and adjacent anatomy. The left panel shows the occlusal view of the maxillary arch, and the right panel shows the panoramic view. CBCT: Cone-beam computed tomography

**Figure 16:**
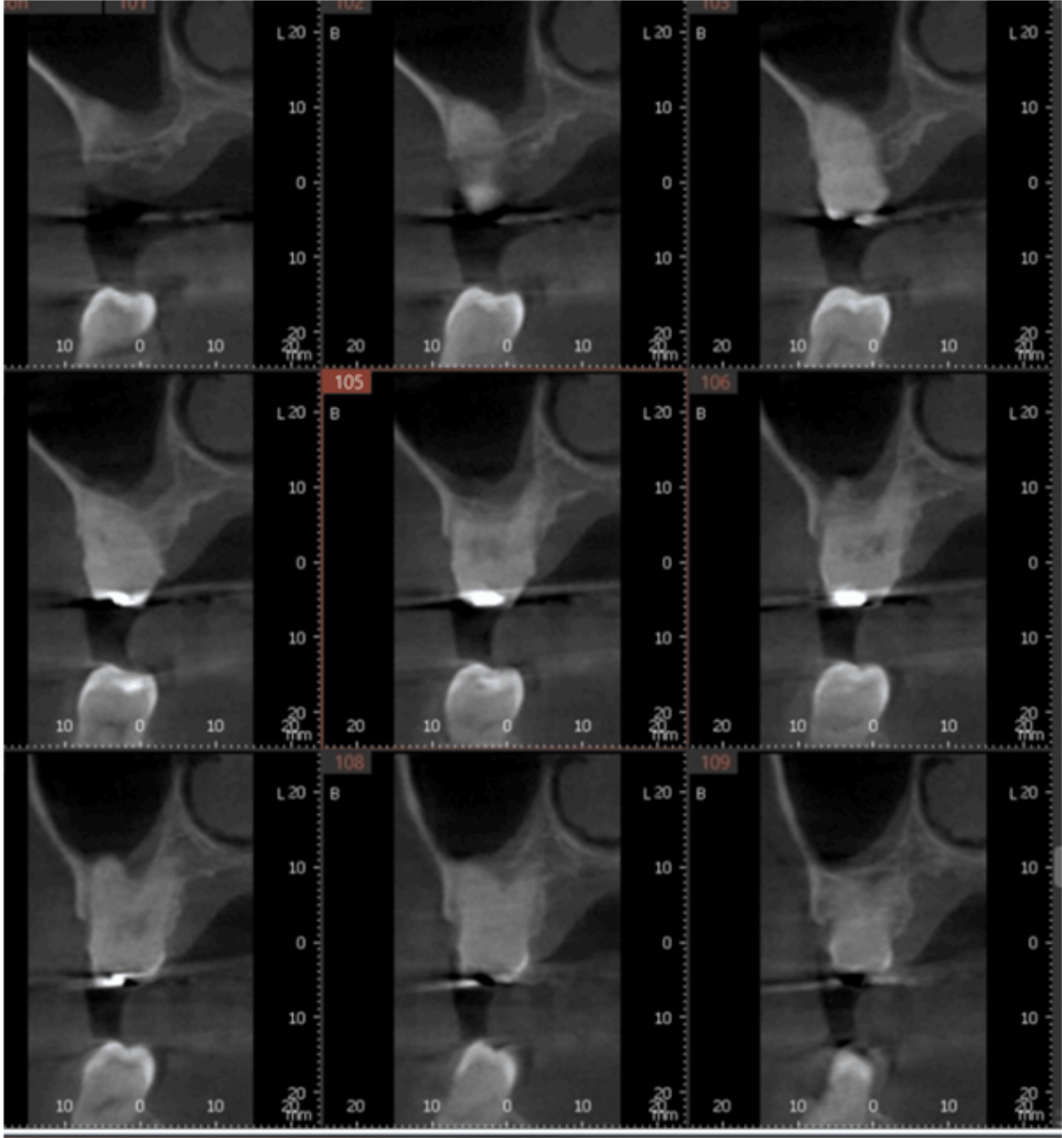
CBCT cross-section of the first molar demonstrating its relation to the maxillary sinus. CBCT: Cone-beam computed tomography

A CBCT scan was performed to evaluate the maxillary dentition, sinuses, and other anatomical structures to facilitate implant planning (Figures 15-16). The analysis of the CBCT indicated that minimal bone was available due to sinus enlargement for the placement of implants into the palatal socket of either the right first or second molars. The palatal root was associated with minimal bone on its buccal surface, and attempting implant placement into that root socket would risk sinus perforation; therefore, sinus augmentation would be required for any implants placed at those sites. In reviewing the left molars, it was noted that sufficient bone existed around the palatal root of the first molar (#14) to allow for implant placement in that socket at the time of extraction. A treatment plan was formulated for the edentulation of the maxillary arch and included implant placement into the right pterygoid area. Angled implants would be placed bilaterally to avoid the maxillary sinus at the first premolar sites, following the anterior sinus wall, with two implants situated between those angled implants and one implant placed in the palatal root socket of the left first molar. The treatment plan was discussed with the patient, and all questions were addressed before he consented to the recommended treatment.

**Figure 17:**
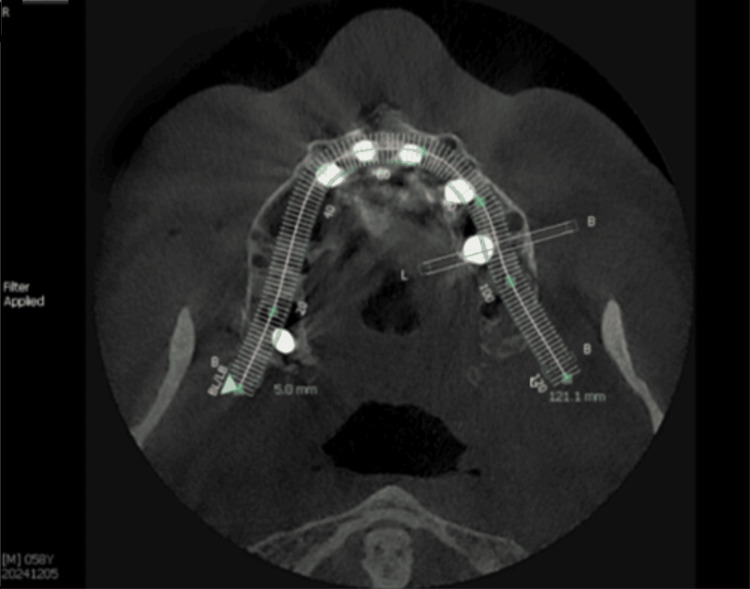
Post-implant CBCT view of the maxillary implants demonstrating implant positioning in relation to the buccal and palatal aspects of the ridge. CBCT: Cone-beam computed tomography

**Figure 18:**
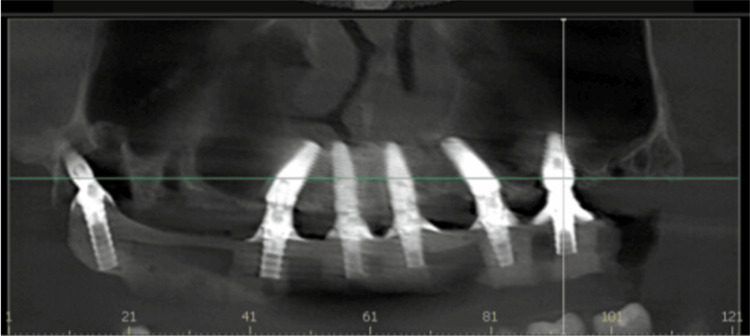
CBCT panoramic view of the implants in relation to the maxillary sinus and surrounding anatomy. CBCT: Cone-beam computed tomography

**Figure 19:**
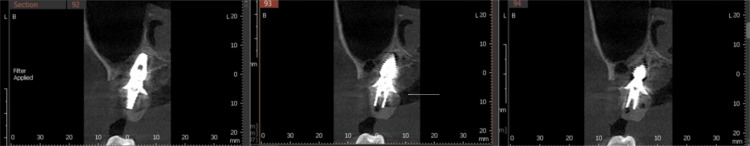
CBCT cross-section of the implant placed into the palatal extraction socket in relation to the maxillary sinus. CBCT: Cone-beam computed tomography

**Figure 20:**
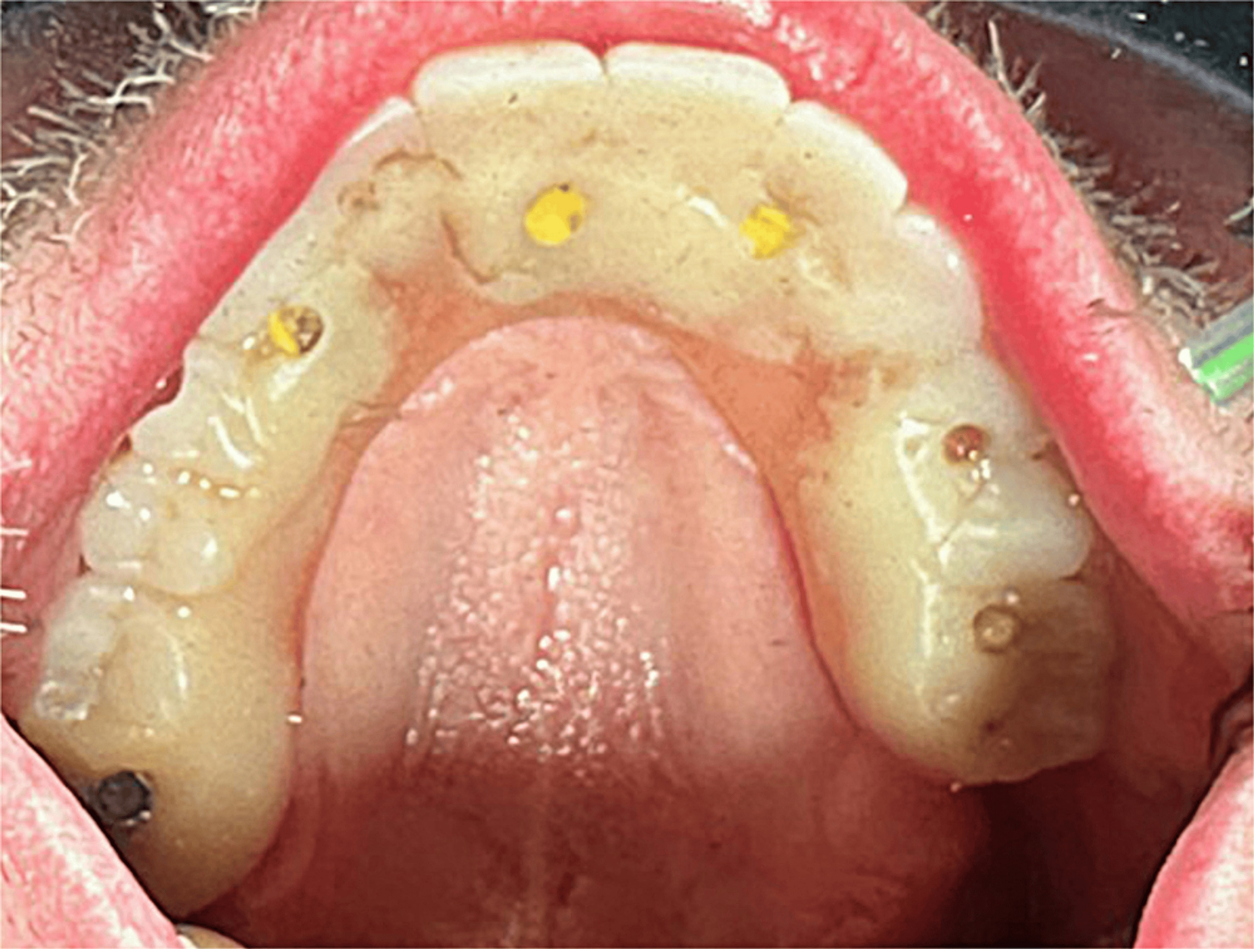
Occlusal view of the maxillary provisional hybrid prosthesis demonstrating the achieved AP spread. AP: Anteroposterior

A similar surgical protocol to that of the previously discussed patient was followed, and implants were placed as follows: #1 (4 mm x 15 mm) (Deep Conical Pterygoid Implant, Southern Implants, Jupiter, FL, USA) with a 3 mm height 30° MUA (Southern Implants, Jupiter, FL, USA); #6 (5 mm x 10 mm) (External 24° Co-Axis® Implant, Southern Implants, Jupiter, FL, USA) with a 3 mm height 0° MUA (Southern Implants); #8 (5 mm x 10 mm) (External 12° Co-Axis® Implant, Southern Implants, Jupiter, FL, USA) with a 2 mm height 0° MUA (Southern Implants); #10 (5 mm x 10 mm) (External 12° Co-Axis® Implant) with a 2 mm height 0° MUA; #12 (5 mm x 10 mm) (External 24° Co-Axis® Implant) with a 3 mm height 0° MUA; and #14 (6 mm x 9 mm) (External Max® Implant, Southern Implants, Jupiter, FL, USA) with a 2 mm height 0° MUA. Insertion torque was 50 Ncm for all implants. An immediate provisional hybrid prosthesis was retrofitted to the temporary titanium abutments (Southern Implants, Jupiter, FL, USA). A CBCT was taken to document the implant placement relative to the patient’s anatomy (Figures 17-18). The implant at site #14 was within the available bone, with bone noted on the buccal aspect of the implant between it and the maxillary sinus (Figure 19). The anteroposterior (AP) spread was maximized by utilizing posterior implant placement in the palatal root of the left first maxillary molar and the right pterygoid positions, as observed on the occlusal view of the provisional hybrid prosthesis (Figure 20).

## Discussion

The utilization of available bone in the posterior maxilla for the palatal root extraction socket at the time of molar extractions is an innovation over traditional methods, which would require sinus augmentation to place an implant at the center of the crest. This technique shortens treatment time, which would be increased when sinus augmentation is utilized, as immediate loading of the posterior implants placed in that area is often not achievable due to insufficient insertion torque. Pterygoid implants may be utilized to bypass the molar area, thereby eliminating a posterior cantilever on the prosthesis. However, the placement of pterygoid implants has potential complications, which can include bleeding, edema, infection, as well as trismus, pain, and neurosensory disturbances [12]. There is also potential for implant displacement into the pterygoid fossa or sinus membrane perforation in atrophic maxillary cases [13]. The utilization of implants placed into the palatal root extraction socket avoids those potential issues and is a simpler surgical procedure. Additionally, impressions are easier to take than when capturing an implant positioned at the very posterior of the arch. The technique described offers a simpler approach to All-on-X when the extraction of the molars is part of the treatment plan for achieving a full arch prosthesis. This approach provides the patient with an easier treatment process, decreases potential complications, and allows for immediate provisionalization of the arch, which may not be possible should sinus augmentation be required for posterior implant placement.

There are potential anatomical risks that, with proper planning and execution of implant placement, can be avoided. The utilization of osseodensification drills, as described, can eliminate or decrease the potential for displacement of the implant into the sinus during placement due to the maintenance of the bone surrounding the palatal root socket [14]. Standard osteotomy drills decrease the socket wall thickness, increasing the potential for implant displacement. Additionally, case selection through analysis of CBCT cross-sections can allow for avoidance of this technique when extensive pneumatization of the sinus between the roots of the molar presents with thin bone in the furcation zone.

The lead author has utilized implant placement in the palatal extraction socket as outlined in 11 patients since 2021, with a total of 17 palatal root implants placed. No failures were noted in those patients. In most cases, 5.0 mm diameter or 6.0 mm diameter implants (occasionally a 7.0 mm diameter max implant) were utilized. Of the cases treated by the author over the past four years, no implant failures were noted, nor were any prosthetic complications reported. Additional studies are recommended to accumulate longitudinal data to further support this technique.

## Conclusions

The treatment of the maxillary arch when edentulation of the posterior teeth is indicated to perform an All-on-X approach can present clinical challenges. These challenges may relate to insufficient crestal height for immediate implant placement in the molar sites due to sinus enlargement. Traditionally, this has been addressed by augmenting the maxillary sinus, often using a lateral approach to create sufficient crestal height for implant placement. This process leads to treatment delays while the sinus graft matures and converts to host bone before implants can be placed. Pterygoid implants have been utilized as an alternative to eliminate a posterior cantilever on the full arch prosthesis, bypassing the enlarged sinus area at the first and second molar sites. However, the use of pterygoid implants presents potential complications, as outlined.

Utilizing the bone surrounding the palatal root of the molar during extraction allows for immediate placement of an implant into that denser palatal bone. This permits immediate loading in a hybrid full arch prosthesis using osseodensification techniques, achieving the recommended insertion torque while avoiding delays associated with lateral sinus augmentation. Placing an implant at the first molar in the palatal root socket eliminates the cantilever that would occur without the placement of a pterygoid implant, thereby avoiding overloading of the implants in the premolar area and anterior to it.
